# Prospective associations between ECG abnormalities and death or myocardial infarction in a cohort of 980 employed, middle-aged Swedish men

**DOI:** 10.1186/s43044-020-00114-9

**Published:** 2020-10-30

**Authors:** Lennart Dimberg, Bo Eriksson, Per Enqvist

**Affiliations:** 1 Department of Public Health and Community Medicine, the Sahlgrenska Academy, University of Gothenburg, Box 454, SE-405 30 Gothenburg, Sweden; 2 Department of Health Metrics, the Sahlgrenska Academy, University of Gothenburg, Gothenburg, Sweden

**Keywords:** Mortality, Myocardial infarction, Cohort study, Middle-aged men, Risk factors, Resting ECG

## Abstract

**Background:**

In 1993, 1000 randomly selected employed Swedish men aged 45–50 years were invited to a nurse-led health examination with a survey on life style, fasting lab tests, and a 12-lead ECG. A repeat examination was offered in 1998. The ECGs were classified according to the Minnesota Code. Upon ethical approval, endpoints in terms of MI and death over 25 years were collected from Swedish national registers with the purpose of analyzing the independent association of ECG abnormalities as risk factors for myocardial infarction and death.

**Results:**

Seventy-nine of 977 participants had at least one ECG abnormality 1993 or 1998.

One hundred participants had a first MI over the 25 years. Odds ratio for having an MI in the group that had one or more ECG abnormality compared with the group with two normal ECGs was estimated to 3.16. 95%CI (1.74; 5.73), *p* value 0.0001.

One hundred fifty-seven participants had died before 2019. For death, similarly no statistically significant difference was shown, OR 1.52, 95%CI (0.83; 2.76).

**Conclusions:**

Our study suggests that presence of ST- and R-wave changes is associated with an independent 3–4-fold increased risk of MI after 25 years follow-up, but not of death. A 12-lead resting ECG should be included in any MI risk calculation on an individual level.

**Supplementary Information:**

The online version contains supplementary material available at 10.1186/s43044-020-00114-9.

## Background

The World Health Organization (WHO) claims that cardiovascular diseases mainly heart attacks and stroke are responsible for more than half of the deaths in Europe, and that 80% of premature heart disease and stroke events are preventable [1]. Traditional risk factors for heart disease include male sex, high blood pressure, tobacco smoking, diabetes, obesity, abnormal blood lipids, and physical inactivity. The Framingham Heart Study started in 1948 and was early to identify and recognize the importance of these risk factors, some of which were recognized already by Galen (150 AD) [2, 3]. The Multinational MONItoring of trends and determinants in CArdiovascular disease (MONICA) have used them to explain national variations of heart attacks and stroke at a population level [4].

ECG is a common investigative method at regular health examinations as well as upon symptoms from the heart such as palpitations and chest pain. A British study of 4739 apparently healthy individuals found ECG abnormalities in 32 % of the middle-aged men [5]. In a systematic review of diabetes patients, Jonas et al. (2018) identified zero randomized controlled studies with resting ECG and cardiovascular outcomes. However, based on findings from five cohort studies, they found small improvements in risk classification for prediction of cardiovascular outcomes by adding ECG to traditional risk factors [6]. Based on this review, the US Preventive Services Task Force (USPSTF) recommends against screening with ECG among apparently healthy primary care patients. They do however recommend screening for those with other concomitant cardiovascular risk factors (intermediate or higher risk).

In 1993, a joint health project, the Coeur study, between a French and a Swedish automotive company, investigated 1000 randomly selected middle-aged French men and Swedish men with focus on risk factors for cardiovascular disease [7]. A major cause was to look into the French paradox of coronary heart disease (CHD). This paradox means that Frenchmen in general, in spite of a less healthy lifestyle with more smokers and intake of cheese and wine, supposedly are less prone to myocardial infarction (MI) compared to men from the Northern countries, for example, Sweden. The aforementioned has been suggested from the MONICA studies [8, 9]. In 1998, we offered a redo of the baseline investigation and several reports have been published looking at the association between the Framingham Risk Index, a common composite risk index for MI, and marital status, life events, and psychological well-being [10–12]. Additional publications from the early phase of the Coeur study concern national differences of carotid artery wall structure between Swedish and French men as well as the association of different indices of overweight and the FRI [13, 14]. In 1999, the international collaboration was terminated, and our project has since focused solely on the Swedish cohort. More recently (2019), Dimberg et al. investigated the association between traditional risk factors and MI and death based on 22 years of accumulated endpoints in terms of first MI and death in the Swedish men [15]. This study confirmed the association of traditional risk factors, specifically with the Framingham Risk Index, and MI, OR of the fifth to first quintile of 23 (95%CI = 5.42, 96.9) but weaker with all-cause death, OR 3.2 (95%CI = 1.65, 6.08). However, traditional risk factors only explained 22% of the variation of MI.

Consequently, we have reason to believe that other independent risk factors possibly such as abnormalities in 12-lead resting ECG may add to this explanation.

This study is a follow-up of the cohort of Swedish men with focus on specific ECG abnormalities and their subsequent associations with myocardial infection (MI) and/or death.

The long follow-up period (now 25 years) and the baseline documentation of a multitude of traditional and non-traditional risk factors allow for the additional risk estimate of ECG abnormalities in a relatively healthy male working population. We therefore expect our findings to benefit the risk prediction of abnormal ECGs at routine health examinations.

The aims are as follows:

- To investigate statistical associations between ECG abnormalities (ECGA) found in resting ECG and the associated risk of death or MI over 25 years
- To calculate the possibly added value of ECGA to the Framingham index for prediction of death and MI throughout this period

## Methods

This study pertains to the Swedish cohort of 1000 randomly selected Swedish employed Caucasian men born between January 1, 1943, and January 1, 1948. They are part of the Coeur project, a prospective longitudinal study started in 1993. At that time, information was collected at a nurse-led health examination and included fasting laboratory data, body measurements, resting blood pressure, pulse, and a 12-lead ECG. The nurse or assistant also supervised the completion of a self-administered questionnaire with questions on health, lifestyle, diet and alcohol intake, mental health, working conditions, stress, and more. This part of the study is detailed in the paper by Simon et al. (1997) [7]. In this research, we have estimated the cardiovascular risk by the Framingham Risk Score. It calculates an individual’s 10-year risk for cardiovascular events. The Framingham Risk Index (FRI) is validated for Americans, and subgroups of Americans, both gender, European Americans, and African Americans [10]. The index was calculated in steps according to Andersson [16].

We used the following baseline data for the present analysis: age; married/single/divorced; smoking status (yes/no); systolic blood pressure (SBP) in mmHg; pulse rate in beats/min; serum high-density lipoprotein cholesterol (HDL-C) in mmol/L; serum triglycerides (TG) in mmol/L, diabetes mellitus (yes/no); left ventricular hypertrophy (yes/no); Framingham Risk Index; alcohol intake in grams/week; body mass index (BMI) in kg/m^2^; and sagittal abdominal diameter in centimeters and ECG. Smoking habits were analyzed by the question about current smoking (yes/no) defined as “Do you presently smoke?”

### ECG collection and analysis

In 1993, the 12-lead resting ECGs and laboratory tests were performed by two nurses (one in Gothenburg and one in Trollhättan) together with supervision of the survey completion. All ECGs were read and coded by the same laboratory technician [7]. The ECG investigation was conducted under a strict protocol, developed by professor Sverker Jern. All ECGs were classified by The Minnesota Code Classification System for Electrocardiographic Findings (MC); see Table 1 [17].

**Table 1:** An overview of the Minnesota Code Classification System for Electrocardiographic Findings

Minnesota Code	ECG abnormality
1-1-1….1-3-6	Q waves
2-1……3-5	QRS axis deviation
3-1……3-3	High amplitude R waves
4-1-1…4-4	ST junction (J) and segment depression
5-1…..5-4	T wave items
6-1…..6-8	A-V conduction defect
7-1-1…7-8	Ventricular conduction defect
8-1-1…8-9	Arrhythmias
	Miscellaneous including ST-segment elevation (9-2)

In 1998, all 1000 Swedish participants were summoned for another nurse-led health check-up including a new ECG. We used the 1993 ECG protocol and the previous laboratory technician (ML) classified each ECG according to MC.

### The 25-year follow-up, end-point information

In 2019, after a new ethical approval, we contacted The National Board of Health and Welfare (Socialstyrelsen), to gain access to the Cause of Death Register data for our 1000 participants and recorded cause of death according to the International Classification of Diseases (ICD) manual. Furthermore, we contacted the Swedish national myocardial infarction registry, also known as Swedeheart to obtain information about heart attacks in our cohort. The registry collects information from all Swedish hospitals that care for patients with acute coronary artery disease and all patients undergoing coronary angiography, catheter intervention, or open-heart surgery [18].

The Joint ESC/ACCF/AHA/WHF task force has described myocardial infarction as myocardial cell death due to prolonged ischemia, and it is a consequence of coronary artery disease [19]. To define myocardial infarction, we used code 410 of the International Classification of Diseases Ninth Revision (ICD 9), and codes I21-I23 of the Tenth Revision (ICD 10) by World Health Organization [20].

### Statistical analysis

The cumulative incidence from baseline to the end of follow-up was estimated as a percentage with confidence limits estimated using the conventional method. Confidence intervals were estimated using exact probability methods when requested due to small numbers. To study the associations between ECG abnormalities (ECGA) and other risk factors by category, we used conventional means with confidence limits, again with exact methods when necessary. To study different models with the dichotomous variables Death or MI as a dependent variable and different combinations of risk factors as independent variables, logistic regressions were used. All such models except one included the dichotomous ECGA. One model contained only the FRI variable as independent. Continuous variables like FRI, SBP, and BMI were represented in the model with dummy variables for the five quintiles of the variable distribution. The results considered in the logistic models were the odds ratios (OR) and a Pseudo-R2. The odds of an event is the risk for the event divided by 1 minus the risk, i.e., the probability for the event divided with the probability for non-occurrence. If the risk is low, we can say that the OR is a fair approximation of the risk ratio (RR) or relative risk. The estimates of OR are given with appropriate confidence intervals.

The logistic regression uses a generalized linear model. We cannot use the traditional R2 to assess the goodness-of-fit of the model. Several Pseudo-R2 have been proposed. The one used here is the one proposed by Mc Fadden [21] that can be interpreted similarly to the R2 for a linear model. The Pseudo-R2 results are expressed as percentages in this paper. Calculations were performed using the software Stata version 15.

Variable definitions, outcomes, and endpoints (dependent variable) include:

- Death 157, deaths reported First Myocardial Infarctions, 100 MI reported

### Explanatory (independent) variables, components of Framingham Risk Index

Explanatory variables include systolic blood pressure (mmHg), smoking (yes/no), HDL cholesterol/Tot cholesterol, left ventricular hypertrophy (yes/no), age, and diabetes (yes/no).

### Additional explanatory variables

Additional explanatory variables include diastolic blood pressure (mmHg), heart rate (beats/min), triglycerides (mmol/l), glycemia (mmol/l), body mass index (BMI), waist/hip ratio, sagittal diameter (cm), total alcohol consumption (g/week), blue collar workers (yes/no), married or cohabiting (yes/no), noisy work environment (yes/no), hypertension (yes/no), family heart attack (yes/no)

## Results

### The study participants and ECG outcome

The sample selected for the baseline survey included 1000 men aged 45–50 years. Of these, 3 did not provide any information. Another 20 had substantial numbers of missing values; particularly, they did not have results from any of the two ECG investigations in 1993 and 1998 (Table 2). Totally, 108 participants had only one ECG, 72 in 1993 and 36 in 1998. ECG means electrocardiogram. ECGA means electrocardiogram with abnormality.

**Table 2:** Electrocardiographic abnormalities (ECGA) of the 977 cohort participants, men aged 45–50, in 1993 (baseline) and at follow up in 1998

	ECG 1998			
ECG 1993	ECGA no	ECGA yes	Missing	Total
ECGA no	790	29	72	891
ECGA yes	19	15	7	41
Missing	36	9	(23)^a^	45
Total	845	53	79	977 (1000)^b^

Classification of ECG outcomes

The 977 participants were classified into three ECG categories as follows: ECG category 0, ECG performed in 1993 and 1998, no ECGA, (*n* = 790); ECG category 1, ECG performed in 1993 and/or 1998, one or more abnormalities (*n* = 79); and ECG category 9, ECG performed in 1993 or 1998 without abnormality (*n* = 108)

^a^23 non-informative

^b^1000-23 informative

As shown in Table 3, surprisingly, category 9 with only one registered and normal ECG compared to category 0 with two normal ECGs had a significantly higher risk of death, but not of MI. As expected, category 1 with one or more ECG abnormalities had over double cumulative percentage than category 0.

**Table 3:** Estimated cumulative incidences (percentages) and absolute numbers of deaths and first MI for participants followed for 25 years by category (95% confidence intervals in brackets)

ECG category	Death (*n* = 157) Cumulative percentage	Infarction (*n* = 100) Cumulative percentage	Deaths Number of cases	First MI Number of cases
0 (*n* = 790)	13.9 (11.5; 16.3)	8.35 (6.42; 10.3)	110	66
1 (*n* = 79)	20.8 (11.5; 30.0)	22.4 (13.8; 31.9)	16	17
9 (*n* = 108)	27.6 (18.5; 36.6)	12.6 (5.83; 19.4)	27	12
Total	15.6 (13.3; 17.57)	10.2 (8.3; 12.1)	153^a^	95^b^

ECG category 0, ECG performed in 1993 and 1998, no ECGA; ECG category 1, ECG performed in 1993 and/or 1998 with one or more abnormalities; and ECG category 9, ECG performed in 1993 or 1998 without abnormality

^a^4 deaths among the 23 non-informatives

^b^5 MIs among the 23 non-informatives

Table 4 only shows statistically significant differences in higher blood pressure (systolic and diastolic) between those with normal ECGs (category 0) and those with ECG abnormalities (category 1).

**Table 4:** Estimated means with 95% confidence intervals in brackets for continuous baseline risk factors by ECG category

Risk factor	ECG category 0 (*n* = 790)	ECG category 1 (*n* = 79)	ECG category 9 (*n* = 108)
Framingham Risk Index	0.089 (0.085–0.093)	0.108 (0.091–0.128)	0.094 (0.080–0.108)
Systolic blood pressure (mmHg)	116 (115–118)	124 (120–128)	116 (113–119)
Diastolic blood pressure (mmHg)	75 (74–75)	79 (76–83)	74 (71–76)
Heart rate (beats per minute)	63 (63–64)	63 (61–66)	62 (61–65)
HDL chol/total cholesterol	0.212 (0.207–0.216)	0.226 (0.197–0.255)	0.207 (0.193–0.222)
Triglycerides (mmol/l)	1.52 (1.46–1.58)	1.53 (1.31–1.74)	1.60 (1.38–1.82)
Glycemia (mmol/l)	5.47 (5.40–5.54)	5.58 (5.27–5.89)	5.39 (5.22–5.57)
Body mass index BMI	25.8 (25.5–26.0)	25.4 (24.6–26.2)	25.0 (24.4–25.6)
Waist/hip ratio	0.933 (0.929–0.937)	0.923 (0.910–0.936)	0.930 (0.919–0.941)
Sagittal diameter (dm)	2.03 (2.02–2.05)	2.02 (1.95–2.08)	1.98 (1.93–2.02)
Alcohol consumption (g/week)	51.5 (47.4–55.6)	52.2 (41.1–63.3)	54.3 (41.7–66.9)

ECG category 0, ECG performed in 1993 and 1998, no ECGA; ECG category 1, ECG performed in 1993 and/or 1998 with one or more abnormalities; and ECG category 9, ECG performed in 1993 or 1998 without abnormality

Table 5 shows a statistically significantly higher percentage of baseline hypertension in those with ECGA (category 1) compared to those with normal ECG (category 0).

**Table 5:** Estimated percentages of “yes” response, with confidence intervals in brackets for dichotomous risk factors by ECG category

Risk factor	ECG category 0 (*n* = 790)	ECG category 1 (*n* = 79)	ECG category 9 (*n* = 108)
Blue collar workers	39 (36–43)	39 (27–50)	36 (26–46)
Married or cohabiting	77 (74–80)	79 (69–88)	74 (65–83)
Noisy work environment	3.7 (2.4–5.0)	8.0 (1.7–14.3)	5.3 (0.69–9.83)
Smoker	28 (25–31)	26 (16–36)	30 (20–39)
Diabetes at baseline	0.008 (0.001–0.014)	0.039 (0.000–0.084)	0.042 (0.000–0.0832)
Hypertension at baseline	8.8 (6.79–10.7)	21 (11.6–30.4)	9.5 (3.48–15.4)
Family heart attack	23 (20.0–25.8)	29 (18.8–39.9)	27 (18.2–36.4)

ECG category 0, ECG performed in 1993 and 1998, no ECGA; ECG category 1, ECG performed in 1993 and/or 1998 with one or more abnormalities; and ECG category 9, ECG performed in 1993 or 1998 without abnormality

No statistically significant risk difference between category 1 and category 0 was seen for blue collar workers (OR = 1.02; *p* = 0.93).

### Regression models

The results from logistic regression models are presented in Tables 6 and 7. The two tables have identical structure and content. Table 6 gives the results for the estimation in a series of models for the outcome, dependent, variable death whereas Table 7 shows the corresponding for the outcome “first MI.”

**Table 6:** Results for regression models with death as a dependent variable with ECGA and additional independent variables of the 977 cohort participants of men aged 45–50 at baseline in 1993 followed for 25 years

Death model	Independent variable(s)	Estimated OR	95% confidence interval for OR	*p* value for OR	Pseudo-R2 for model, %
ECGA only (model 1)	ECGA = 0	Reference			1.4
	ECGA = 1	1.52	(0.83; 2.76)	0.106	
	ECGA = 9	2.09	1.26 3.46	0.001	
FRI only (model 2)	FRI = 1	Reference			2.9
	FRI = 2	0.68	(0.35; 1.31)	0.248	
	FRI = 3	0.77	(0.40; 1.46)		
				0.418	
	FRI = 4	1.51	(0.66; 2.67)	0.154	
	FRI = 5	2.16	(1.25; 3.73)	0.006	
ECGA and FRI (model 3)	ECGA = 0	Reference			3.8
	ECGA = 1	1.58	(0.85; 2.93)	0.146	
	ECGA = 9	1.84	(1.06; 3.20)	0.030	
	FRI = 1	Reference			
	FRI = 2	0.68	(0.35; 1.32)	0.256	
	FRI = 3	0.69	(0.36; 1.34)	0.272	
	FRI = 4	1.40	(0.75; 2.51)	0.246	
	FRI = 5	2.12	(1.22; 3.69)	0.007	
ECGA and FRI variables (model 4)	ECGA = 0	Reference			8.3
	ECGA = 1	1.41	(0.73; 2.73)	0.301	
	ECGA = 9	1.84	(1.01; 3.37)	0.048	
ECGA and all explanatory variables (model 5)	ECGA = 0	Reference			13.8
	ECGA = 1	1.42	(0.69; 3.02)	0.247	
	ECGA = 9	2.52	(1.27; 4.97)	0.021	

ECG category 0, ECG performed in 1993 and 1998, no ECGA; ECG category 1, ECG performed in 1993 and/or 1998 with one or more abnormalities; and ECG category 9, ECG performed in 1993 or 1998 without abnormality

**Table 7:** Results for regression models with first MI as a dependent variable with ECGA and additional independent variables of the 977 cohort participants of men aged 45–50 at baseline in 1993 followed for 25 years

Infarct models	Independent variable(s)	Estimated OR	95% confidence interval for OR	*p* value for OR	Pseudo-R2 for model, %
ECGA only (model 1)	ECGA = 0	Reference			2.11
	ECGA = 1	3.16	(1.74; 5.73)	0.000	
	ECGA = 9	1.59	(0.82; 3.06)	0.168	
FRI only (model 2)	FRI = 1	Reference			8.57
	FRI = 2	6.35	(1.40; 28.8)	0.017	
	FRI = 3	9.27	(2.11; 40.7)	0.003	
	FRI = 4	14.3	(3.24; 61.5)	0.000	
	FRI = 5	25.4	(6.02; 106)	0.000	
ECGA and FRI (model 3)	ECGA = 0	Reference			10.6
	ECGA = 1	2.82	(1.51; 5.28)	0.001	
	ECGA = 9	1.43	(0.70; 2.89)	0.324	
	FRI = 1	Reference			
	FRI = 2	6.22	(1.36; 28.3	0.018	
	FRI = 3	7.98	(1.79; 35.5)	0.006	
	FRI = 4	14.1	(3.28; 60.6)	0.000	
	FRI = 5	24.3	(5.77; 103)	0.000	
ECGA and FRI variables (model 4)	ECGA = 0	Reference			14.9
	ECGA = 1	3.43	(1.74; 6.76)	0.000	
	ECGA = 9	1.24	(0.57; 2.70)	0.579	
ECGA and all explanatory variables (model 5)	ECGA = 0	Reference			24.6
	ECGA = 1	4.22	(1.85; 9.64)	0.001	
	ECGA = 9	1.18	(0.47; 2.96)	0.719	

ECG category 0, ECG performed 1993 and 1998, no ECGA; ECG category 1, ECG performed 1993 and/or 1998 with one or more abnormalities; and ECG category 9, ECG performed 1993 or 1998 without abnormality

The independent variables in the models 1–5, from top to bottom in the two tables, are:

1. Category of ECG
2. Quintiles of the FRI
3. Category of ECG together with FRI
4. Quintiles of ECG together with the variables that are included in the FRI, here considered separately in linear form
5. Quintiles of ECG, the FRI variables in linear form and the additional; total alcohol, heart rate, BMI, waist/hip ratio, and sagittal diameter

For the two last models, the tables contain only the estimates for ECGA and total model Pseudo R2.

The main interest is the association between death and ECG abnormality. We do not find a statistically significant association in any of the models in Table 6. The lack of statistical significance with OR between 1.4 and 1.6 is largely due to the small number of ECGA. The significant OR comparing the categories coded 0 and 9 is difficult to interpret since the last category is problematically defined.

The Pseudo-R2s are generally low even for the largest model (13.8). It shall be noted though that the ECGA variable has some importance. However, even at the low level, the model with ECGA and the individual FRI variables is better than the model where the calculated FRI is used.

For MI (Table 7), the OR for comparisons of ECGA = 0 and ECGA = 1 are all statistically significant whereas those comparing ECGA = 0 and ECGA = 9 are not. ECG investigation is statistically significantly associated with MI but not with death. The OR for ECGA are all fairly high, between 2.8 and 4.2, regardless of the model.

The R2s are higher for MI than for death and the model with individual FRI is higher than the model with computed FRI, same as for death. The highest R2 is almost 25%, which is higher than 13% for death. Both, however, shall be considered as relatively low.

Tables 8 and 9 show the distributions of ECG abnormalities by types according to the Minnesota classification.

**Table 8:** Number of ECG deviations in 1993 electrocardiographic abnormalities and myocardial infarction and death in 964 middle-aged, employed men in 1993 followed for 25 years

Minnesota Code	Explanation	Investigated persons	Observed findings	Findings with death	Findings with infarct	*p* value death	*p* value infarct	ECG findings both in 1993 and 1998
Q and QS patterns								
1:1	Q and QS pattern (% with endpoint)	964	3	1 (33)	2 (67)		0.000	2
1:2	Q, QS, and QSR pattern (% with endpoint)	964	10	1 (10)	2 (20)			5
STJ and segment depression								
4:1 or 4:2	STJ depression (% with endpoint)	947	5	4 (80)	3 (60)	0.000	0.000	1
T wave items								
5:1 or 5:2	T wave negative or biphasic (% w. endpoint)	947	7	3 (43)	2 (29)	0.004	0.075	3
AV conduction defect								
6:1	Complete AV block	964	0					
6:4:1	Wolf-Parkinson-White pattern	964	0					
6:8	Artificial pacemaker	964	0					
Ventricular conduction defect								
7:1:1	Complete left bundle branch block	964	1	0	0			0
7:2:1	Complete right bundle branch block	964	7	1 (14)	2 (29)			4
7:3	Incomplete RBB	964	23	1 (4)	5 (22)			11
7:4	Intraventricular block	962	11	4 (36)	1 (9)	0.004		0
7:6	Incomplete LBB	919	8	1 (13)	1 (13)			

The *p* values refer to death and infarct risk comparisons between persons with and without ECG abnormalities. Only *p* values smaller than 0.100 are shown. Note that the *p* values themselves are due to variation. Even one single finding more or less might change the *p* value substantially.

**Table 9:** Number of ECG deviations in 1998 follow-up

Minnesota Code	Explanation	Investigated persons	Observed findings	Findings with death	Findings with infarct	*p* value death	*p* value infarct
Q and QS patterns							
1:1	Q and QS pattern	944	15	2 (13)	3 (20)		
1:2	Q, QS, and QSR pattern	942	16	1 (6)	3 (19)		
STJ and segment depression							
4:1 or 4:2	STJ depression	926	18	4 (22)	4 (22)	0.054	0.041
T wave items	T wave negative or biphasic						
5:1 or 5:2		924	19	4 (21)	5 (26)	0.072	0.006
AV conduction defect							
6:1	Complete AV block	945	0				
6:4:1	Wolf-Parkinson-White pattern	945	0				
6:8	Artificial pacemaker	945	0				
Ventricular conduction defect							
7:1:1	Complete left bundle branch block	945	1	0	0		
7:2:1	Complete right bundle branch block	945	9	1 (11)	2 (22)		
7:3	Incomplete RBB	945	26	3 (12)	5 (19)		
7:4	Intraventricular block	944	4	0	0		
7:6	Incomplete LBB	919	5	1 (20)	1 (20)		

The *p* values refer to death and infarct risk comparisons between persons with and without ECG abnormalities. Only *p* values smaller than 0.100 are shown. Note that the *p* values themselves are due to variation. Even one single finding more or less might change the *p* value substantially. Percentages in brackets

Strongest association with MI was Q, QS pattern (1/3 = 33%) (as expected) and STJ depression (4/18 = 22%), but the association with the more common incomplete right bundle branch block (5/23 = 22%) and intraventricular block (1/11 = 9%) were surprising to us, although based on small numbers and therefore not significant at that level.

Also surprising were the strong associations with death of STJ depression (4/5 = 80%) and intraventricular block (4/11 = 36%) with the same caution of interpretation.

**Fig. 1:**
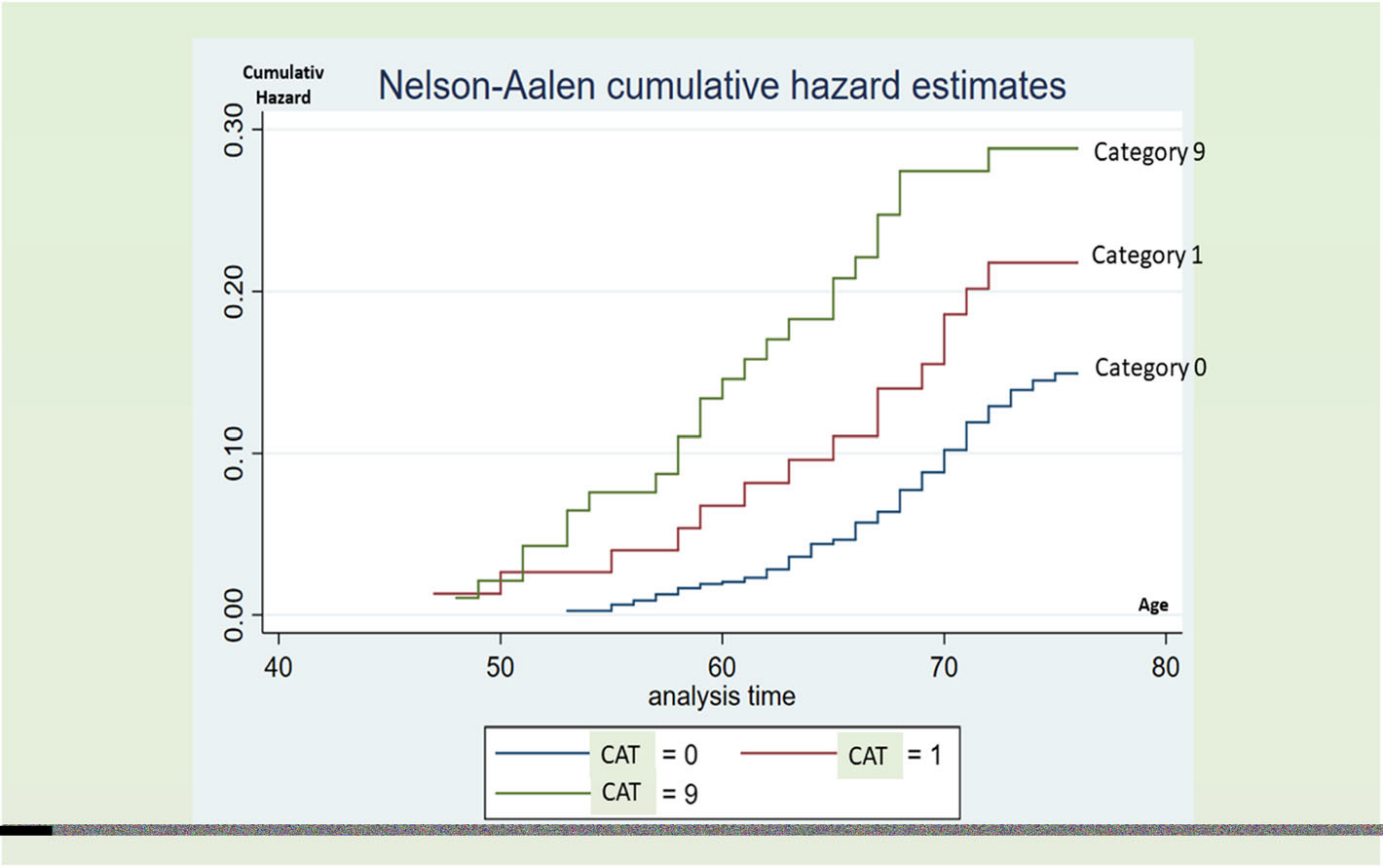
Nelson-Aalen Cumulative Risk Estimates for Death by category in 977 men. Note: ECG category 0, ECG performed in 1993 and 1998, no ECGA; ECG category 1, ECG performed in 1993 and/or 1998 with one or more abnormalities; and ECG category 9, ECG performed in 1993 or 1998 without abnormality

The study started in 1993 when the participants were 45–50 years old and finished in 2018 when they were 70–75 years old. The cumulative deaths, all causes, by age at death in category 9 (only first ECG) compared to category 1 (first and second ECG with at least 1 abnormal) and category 0 (two normal ECGs) indicate that category 9 over 25 years follow-up was at highest risk, as shown in Fig. 1.

**Fig. 2:**
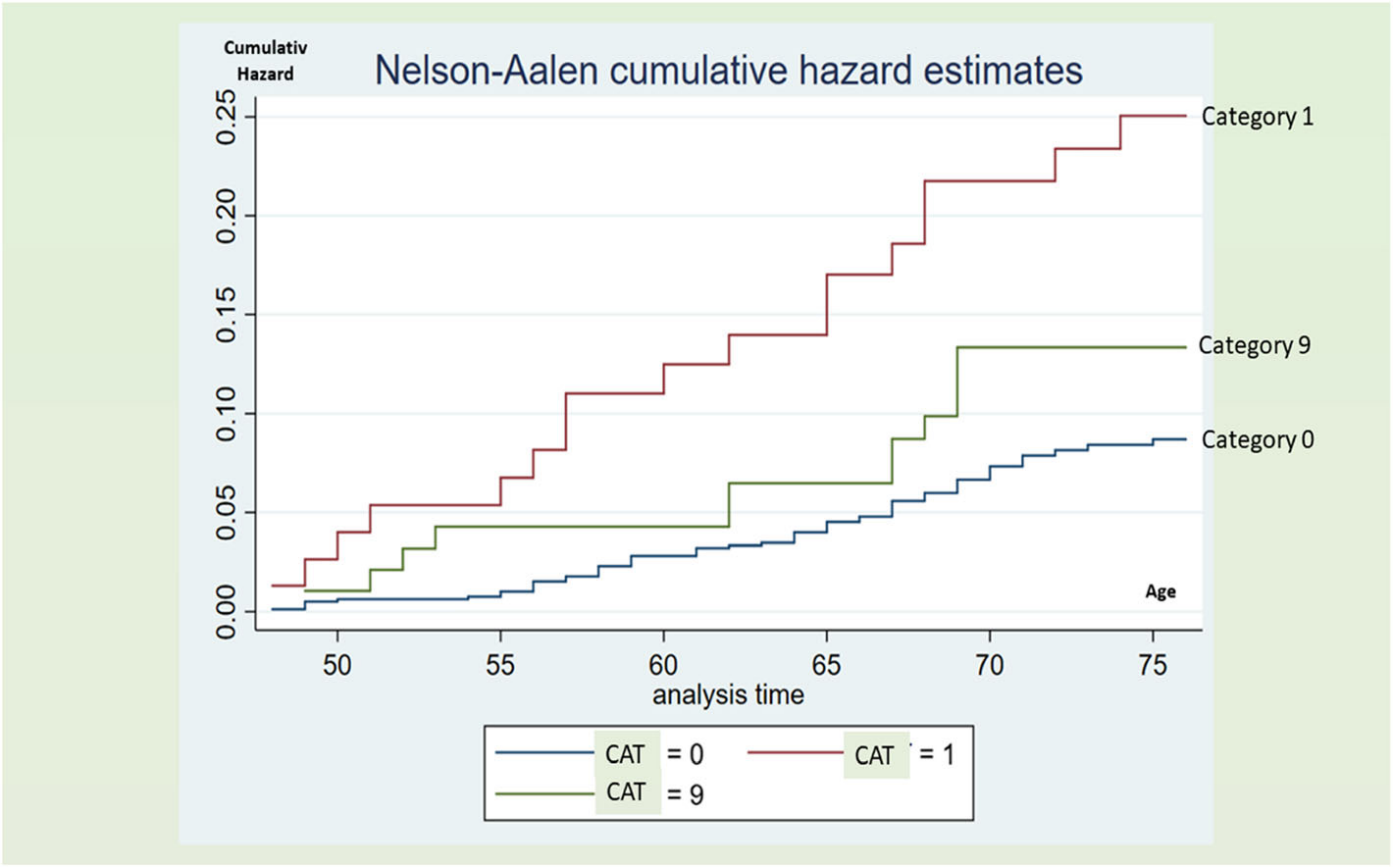
Nelson-Aalen Cumulative Risk Estimates for Myocardial Infarction by category in 977 men. Note: ECG category 0, ECG performed in 1993 and 1998, no ECGA; ECG category 1, ECG performed in 1993 and/or 1998 with one or more abnormalities; and ECG category 9, ECG performed in 1993 or 1998 without abnormality

Contrary to the cumulative deaths and correspondingly, Fig. 2 shows the significant outcome of MIs highest in category 1 (at least one abnormal ECG).

## Discussion

A major finding is that there is a statistically significant association between resting ECG abnormalities (ECGA) at baseline as a group and the cumulative incidence of myocardial infarction over 25 years also after adjustment for traditional risk factors. The ORs quantifying the comparisons are all high, between 2.8 and 4.2, regardless of the regression model used.

The benefit of ECGA compared to traditional risk factors following adjustment indicates that ECGA may not follow the same arteriosclerotic pathway as the Framingham Risk Index.

However, the Pseudo-R2, which explains the proportion of the variation, was for ECGA only 2.1% compared to FRI with 8.6%. The model combining ECGA and FRI has a Pseudo-R2 at 11%, still low.

There is no statistically significant association between ECGA and the outcome of death.

In 2018, based on a review of the literature, the US Preventive Services Task Force (USPSTF) published a recommendation not to use ECG for screening in previously healthy men without any other cardiovascular risk factor [22]. Our study and others though indicate that resting ECGs add value for the predictive model of the risk of future myocardial infarction [6, 23–32].

It is important to realize the difference between using ECG for screening of an assumed healthy population and using it as a tool among others in clinical practice. Using the present data as an example, a population screening would have a sensitivity of 20%, i.e., only one in five would be classified as a potential MI patient. Further, there will be about 75% false positive, unnecessarily requiring some attention. Population screening aims at finding potential cases early and an important requirement also is that there is some action that can be taken, which hardly is the case in the present study.

In clinical work, the assumptions are different with higher sensitivity and less false positive due the higher prevalence of persons with high risk.

It can be clinically relevant to examine healthy 45–50-year-old men with ECG as one component among others when judging the risk for an MI.

Since no remedy exists for unspecific ECG abnormalities, such findings may only be used to emphasize the important prevention of a healthy lifestyle.

Due to the few cases in the subgroups of the ECG abnormalities, it is not meaningful to evaluate statistically the impact of most Minnesota Code subgroups on the specific risk of MI.

Other studies suggest that prolonged QRS complexes (24–27) and negative T-waves (24, 28–29) lead to increased risks. This is not contradicted by our study.

In addition, we also find that STJ depression shows an increased but not statistically significant risk for MI and death.

Prolonged QTc (pulse-corrected QT-time) has been associated with higher risk of MI and death [32–35]. Prolonged QTc is not classified in the Minnesota Code and has therefore not been studied here.

An unexpected finding was that the category 9 participants (only one EGG taken and without abnormality) had a double risk of death, 28% vs 14%, (*p* < 0.05). We have no satisfactory explanation for this discovery. Some participants may have been skeptical or ill and refrained from the second follow-up. One thought was that this phenomenon might have been caused by death between the two examinations (in 1993 and 1998) but as seen in Fig. 2, this is not the explanation.

Another finding shown in Tables 5 and 6 is that there is more information in the variables included in FRI if they are studied separately than in the combined risk index (FRI). The Pseudo-R2 for MI goes from 11 to 15% and for death from 3.8 to 8.3%.

### Limitations and strengths

#### Strengths

The original 1000 men were randomly selected from a well-defined population of male workers followed over a very long time (25 years). Very few rejected to participate. The nurses and health professionals were specially trained and well-defined methods were used. The procedures for baseline information acquisition were carefully developed based on current standards. Accurate information about the endpoints was available through national registers with quality control to contain valid data. The internal validity of the study and its results have throughout been carefully secured.

#### Limitations

The external validity, i.e., validity for other populations with other contextual structures, is clearly limited. The study is confined to males in a certain age range in a particular industry and certain country. The comparably small sample size and, therefore, the small number of outcomes events are limited and are a problem to some extent.

The study is also limited by the use of the Framingham Risk Index in the original Coeur study since this index has been superseded by better predictive tools such as QRISK2 [36] and the use of biomarkers [37]. However, in a 25-year longitudinal study, the analysis is necessarily restricted to the original risk measure. Important to point out is that even QRISk2 at its core include the traditional risk factors from the Framingham study. Another limitation is that the Minnesota Code used for classification does not register QTc.

### Confounding

The main research question concerns the statistical association between the risk and results of the ECG investigations. This relation can be confounded by several variables. Many of these were observed at baseline such as age, smoking status, alcohol consumption, weight, height, and systemic blood measurements. Most of these are correlated with each other and with the risk of MI and death. Therefore, the crude correlation between risk and ECGA is confounded by a manifold variable. Among these, some are known and possible to observe. Others are known but not possible to observe and yet others have not even been imagined. The first mentioned group can be adjusted for using different regression models. For this paper, we have tried several models. An important finding is that the OR for ECG abnormality remains reasonably stable, OR about 3.4, regardless of the model. This is an indication that the main results are not seriously confounded by the variables used in this study.

### Bias

A bias is a systematic error that has similar size and direction for a group of measurements. Several biases could influence the results. For example, laboratory results might be biased due to incorrect calibrations; questions for self-reporting can be formulated to give biased information. We have tried to address some of these biases by careful quality control and the use of certified laboratories in our study.

In longitudinal studies, it is possible for both confounding and biases to occur since it is difficult to account for changes in an individual’s habits or health status over the whole, in this case, 25-year period.

Confounding factors in this study would include not accounting for the eventuality that participants’ behaviors might change over time, for example smoking less, losing or gaining weight as well as medical treatment of cholesterol and hypertension, making the baseline risk factors obsolete in some cases.

Although we do not have secular, longitudinal data of the 25-year period, the findings presented in another longitudinal Swedish study of 50-year-old men followed for 50 years which show secular changes: smoking less, lower cholesterol but higher BMI, and a more sedentary lifestyle in cardiovascular risk factors over time are likely to apply to our cohort [38]. Furthermore, the addition of medication during this period may have changed the prognosis of some endpoints.

## Conclusions

The study showed that there was an about 3- to 4-fold independent risk for a first MI in persons with at least one ECGA compared to those with normal ECGs found in resting ECGs of healthy Swedish working men aged 45 to 50 years over the following 25 years. A 12-lead resting ECG should be included in any MI risk calculation on an individual level.

A parallel statement for death could not be made.

Presence of ST- and R-wave changes are predictors of MI risk but not of death and adds some information of MI risk variation to that which can be obtained using the Framingham Risk Index.

The highest Pseudo-R2 observed in the study is found to be a predictor of a MI model with 20 independent variables. It reaches almost 25%, however, meaning that we must assume that there is still a large number of variables, not observable or totally unknown that influence the risks.

## Supplementary Information

**Additional file 1: Appendix 1**. The Renault-Volvo Coeur project group 1993-1998.

## Data Availability

May be available upon request to the corresponding author.

## Abbreviations

CI: Confidence interval
CVD: Cardiovascular disease
ECG: Electrocardiography
ECGA: Electrocardiography abnormality
FRI: Framingham Risk Index
FRS: Framingham Risk Score
HR: Hazard ratio
ICD: International Classification of Diseases
MC: The Minnesota Code Classification System for Electrocardiographic Findings
MI: Myocardial infarction
OR: Odds ratio
Pseudo-R2: Calculation of how much of the outcome variation is explained by the model
P-Value: Probability value
QRS-complex: An ECG pattern that symbolizes the depolarization of ventricles
QTc: An ECG pattern that measures the time between ventricles depolarization and ventricles repolarization corrected for pulse rate
RR: Relative risk
STJ: ST-junction = an ECG pattern symbolizes phase 2 of ventricular repolarization
T-Wave: An ECG pattern symbolizes phase 3 of ventricular repolarization
USPSTF: US Preventive Services Task Force
WHO: World Health Organization
