# Supplementary material for: Prospective associations between ECG abnormalities and death or myocardial infarction in a cohort of 980 employed, middle-aged Swedish men
Source: Egypt Heart J. 2020 Oct 30;72:75. doi: 10.1186/s43044-020-00114-9 (PMC7599283; doi:10.1186/s43044-020-00114-9)
Supplement: Supplementary file 1 — Additional file 1: Appendix 1. The Renault-Volvo Coeur project group 1993-1998. [file 43044_2020_114_MOESM1_ESM.docx]

**Appendix 1**

The Renault-Volvo Coeur project group 1993-1998

Contributing to the study protocol were Lennart Hansson [1], Björn Dahlöv [1], Per Mårin [1], and Per Björntorp [1]. Project leader for Volvo was Lennart Dimberg assisted by Lars Kumlin [2], and for Renault Catherine Lanoiselée/Guillemette Latscha [3]. Scientific leader was Alain Simon assisted by Jaime Levenson [4]. In Sweden, responsible for the statistical calculations was Bo Eriksson, Nordic School of Public Health [5]. He was helped by Nils Gunnar Pehrsson [6], Henrik Ahlbom [6], and Maria Persson [6].

In France, statistical analyses were provided by Marc Massonneau [7], and Nicolas Denarié [4]. Mats Andrén [1] was instrumental in setting up the software for data input.

Additional contribution from Broussais Hospital came from Jérôme Gariépy, and Gilles Chironi [4]. Lena Rossander-Hulthén [1] developed the Diet Study Protocol. Alain Simon [4] was responsible for the Ultrasonography Study Protocol in cooperation with Marc Massonneau [7], and Sverker Jern [1]. Jaime Levenson [4] was responsible for the Blood Viscosimetry Protocol together with Lena Sjögren, Astra-Zeneca. All blood hormone analyses were performed by Carola Gustafsson [1] and all EKG analyses were performed by Margareta Leijon [1]. Kristina Orth-Gomér [8] was involved in the design of the psychological questions, and advice in particular of step 2 and 3, as well as in the writing of several papers and the tutoring of Lars Kumlin [2]. Leif Wallin [9] and Gisela Rose [9] contributed to the design of the psychosocial questions and analysis. Several physicians at Volvo particularly Carl-Erik Hedström [9] and Irma Wright [9] and at Renault, particularly Madeleine Leroy, Olivier Galamond, Christine Morvan, Sylvie Selosse and Jacques Sissler [3], have contributed to the study.

Richard Sloan [10] and Thomas Bigger [10] designed the Holter EKG protocol and analyses. Holter EKG was performed on Swedish men only.

The baseline investigations at Volvo were performed by Siv Thornell [2], Lisbeth Paffrath [9], and Pia Johannisson [9]. The second step investigations at Volvo were conducted by Siv Thornell [2], Kerstin Terning (dietician) [1], Lillemor Engström, Lab tech. (Swedish viscosimetry) [11].

Christer Erkenborg [2] was instrumental for the practical and technical arrangements at Volvo Aero Corporation.

The third step interviews were conducted by Siv Thornell [2], Annie Jansson [1], Caroline Karlsson , Inga-Greta Wittlöv, and Pia Lindén.

Xiaodong Cai [12] at the World Bank, Washington, DC contributed to statistical analyses and results from two of the studies.

The study benefited from the professional secretarial assistance of Britt Lindström [2], and Maria Ortega [4], and the financial coordination of Marianne Berlin [2], and Kenneth Schultz [2].

Sandra Ross [12] and Lorraine Nagy [12] assisted with language revision.

The project was financed entirely by the Volvo and Renault enterprises. Last but not least I wish to thank the Director of the World Bank Health Services Department Bernard Demure, who not only was involved in the early planning of this project, then working for the Renault enterprise, but also allowed the Coeur project to be part of Lennart Dimberg’s work program at the World Bank.

*All affiliations are given at the time of the study initiation, or publication*

[1] Sahlgren’s/Östra/Vasa hospital, Gothenburg, Sweden

[2] Health Department,Volvo Aero Corporation, Trollhättan, Sweden

[3] Service Medical, Renault Automobiles, Billancourt, France

[4] Broussais Hospital, Paris, France

[5] Nordic School of Public Health, Gothenburg Sweden

[6] Statistiska konsultgruppen, Gothenburg, Sweden

[7] IODP, Paris, France

[8] Department of Preventive Medicine, Karolinska Institute, Stockholm, Sweden

[9] Volvo, Gothenburg, Sweden

[10] Columbia-Presbyterian Medical Center, New York

[11] Norra Älvsborgs Länssjukhus, Trollhättan, Sweden

[12] The World Bank, Washington DC, USA
